# Obstacle Detection in Infrared Navigation for Blind People and Mobile Robots

**DOI:** 10.3390/s23167198

**Published:** 2023-08-16

**Authors:** Ioannis Papagianopoulos, Gilbert De Mey, Andrzej Kos, Boguslaw Wiecek, Vasilis Chatziathasiou

**Affiliations:** 1 Department of Electrical and Computer Engineers, Aristotle University of Thessaloniki, 54124 Thessaloniki, Greece; ioannis.papagianopoulos@auth.gr (I.P.); hatziath@auth.gr (V.C.); 2 Department of Electronics and Information Systems, Ghent University, Technologiepark 126, 9052 Ghent, Belgium; gilbert.demey@ugent.be; 3 Institute of Electronics, AGH University of Science and Technology, al. Mickiewicza 30, 30-059 Krakow, Poland; 4 Institute of Electronics, Lodz University of Technology, ul. Wólczańska 221, 90-924 Lodz, Poland; wiecek@p.lodz.pl

**Keywords:** corner navigation, blind people, mobile robots, infrared sensors

## Abstract

The paper is a continuation of the authors’ work intended for infrared navigation for blind people and mobile robots. This concerns the detection of obstacles in the person’s or mobile robot’s trajectory, in particular, the detection of corners. The temperature distribution of a building’s internal wall near a corner has been investigated. Due to geometry, more heat will be transferred by conduction so that inside the building, the temperature on the wall will be decreasing towards a corner. The problem will be investigated theoretically and numerically, and the results are confirmed by experimental measurements. The purpose of this research is to help blind people by equipping them with a small infrared camera that warns them when they are approaching a corner inside a building. The same aim is addressed to mobile robots.

## 1. Introduction

Nowadays, there are more than 250 million visually impaired people worldwide, including blind people [1,2]. There are many commercial products enabling blind people to navigate in different environments. Most of these solutions are based on pattern recognition with the use of a camera operating in the visible range [3,4,5]. However, the visible range requires filtering out the different images that appear on the path being navigated. This delays information processing and also increases system power consumption. It should be emphasized that the original use of infrared eliminates this disadvantage.

**Figure 1:**
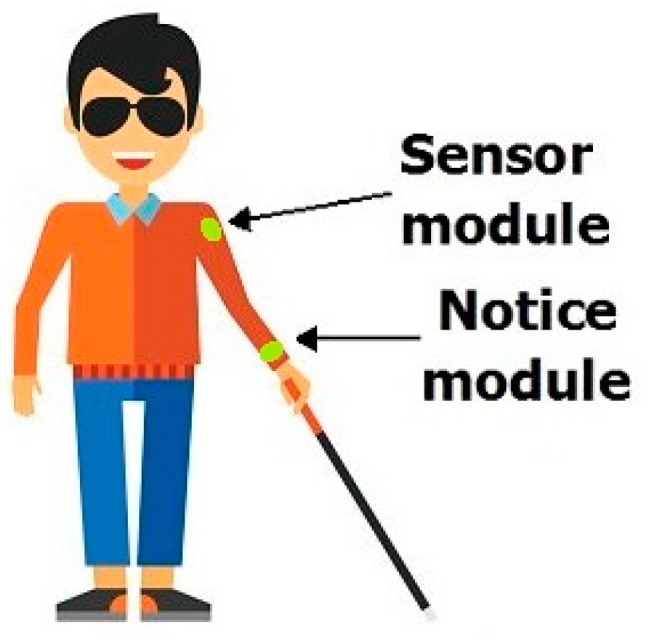
IR sensor and data processing system on arm [17].

Another proposal is the use of ultrasonic or laser sensors for obstacle location [6,7]. In both cases, the generation of a measurement signal is required, which consumes energy. In our original idea, the measurement signal is the heat radiation always present in the environment, available for free. This is of particular importance for mobile systems where energy saving is critical. These above-mentioned solutions allow to facilitate the navigation of blind people in indoor and outdoor circumstances. Currently, there are several solutions on the world market that can detect pedestrian crossings. These solutions assist in avoiding pedestrian–car collisions and usually are based on the video camera mounted on the car [8,9]. An interesting solution is human detection during the night using infrared cameras [10,11,12]. The main difficulty for blind or visually impaired people is navigating between selected places due to the presence of various obstacles. Although there are more and more additional facilities on paths to help blind people navigate, most roads or streets, as well as corridors, are currently devoid of such solutions. These solutions are quite costly and require changes in the urban infrastructure. The authors proposed a personal navigation system which enables movement indoors and outdoors. Our solution uses an infrared sensor attached to the person’s arm, Figure 1. 

The modern development of IR equipment allows the use of cheap IR cameras [13,14], which additionally makes our method useful and easy to implement.

The thermal information on the detected pedestrian crossing is signaled through vibrations. Our method is the next step in the efforts aimed at constructing a complex system for the navigation of blind people and mobile robots, especially inside buildings (galleries, office blocks, etc.) where GPS navigation is not available [15]. The solution makes it easier to move along the selected object (e.g., a wall or shop window) at a specified distance. 

The method proposed in the articles can support the navigation of a blind person in places where there are no special facilities for the visually impaired. Additionally, the proposed solution described in this paper enables the detection of corners, which is a serious obstacle for blind people or robots walking along a wall.

Heat transfer through the wall of a building is governed not only by conduction in the solid material but also by convection at the inner and outer sides of the wall. If the wall is made from solid bricks, the thermal resistance due to convection becomes comparable to the value due to thermal conduction. It means that a non-negligible temperature drop will be built up between the air and the walls’ surface. In the area around a corner, the phenomenon will be even more pronounced.

The use of infrared cameras is implemented in some cars to improve navigation [11]. But in recent years, the price of infrared cameras has reduced a lot, mainly due to the introduction of microbolometer detectors. Nowadays, it is possible for a private person to purchase such equipment. This can provide serious help for blind people, as has been described in a few publications. 

This article is an extension and supplement to our work on a comprehensive system supporting the movement of blind people and robots inside buildings [15,16,17,18]. The infrared sensor is fixed at the top of the blind person’s arm. All hardware and software are analogous to mobile robots. The acquired signals are sent to the Notice module, where they are processed into information humans can understand, i.e., vibrations. This solution is intended to help blind people navigate without the use of an acoustic signal that negatively affects the perception of the environment [17], Figure 1.

**Figure 2:**
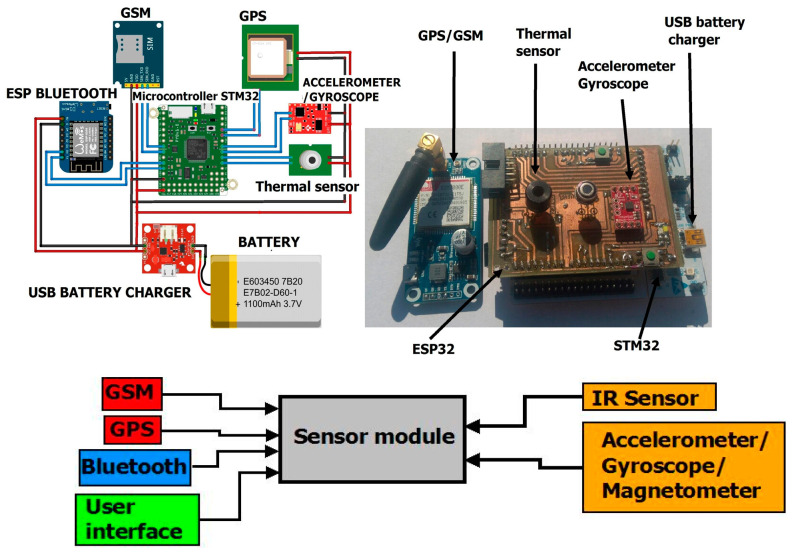
Sensor module [17].

**Figure 3:**
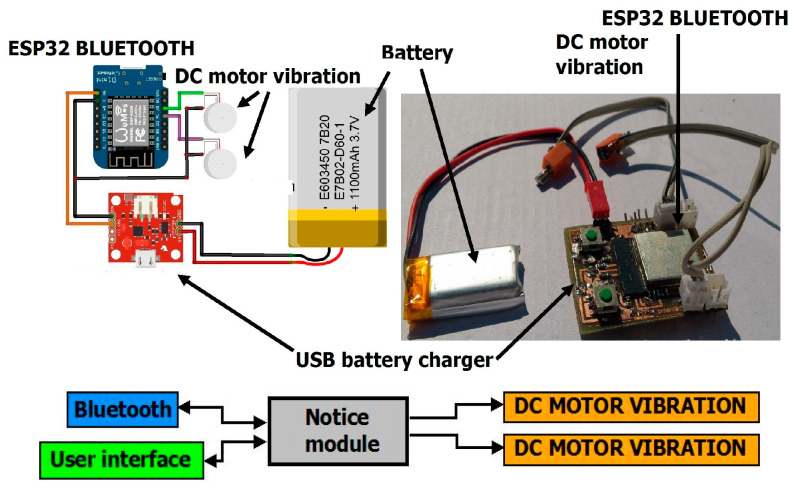
Notice system [17].

The swinging of the infrared sensor in time with the movement of the hand is eliminated using a numerical algorithm. The appearance of the Acquisition electronic system as well as the Notice system are shown in Figure 2 and Figure 3. 

This article presents an extension of our electronic system to recognize the approaching obstacle in the form of the end of the corridor. It allows people to recognize whether it is the end of the building or only its internal corner. In the case of the inner end of the corridor, there will be no temperature difference, which is important information for a human or robot. Such an obstacle will be detected by our method using the difference in emissivity of floors and walls, which is described in our work [17].

More specifically, the article presents how a thermal camera will warn a blind person or a robot when he/she/it approaches a corner inside a building, which marks the end of the building. The authors paid special attention to the practical aspect of using the results to implement a mobile security system. This paper contains both a theoretical and an experimental overview of our proposal extending the possibilities of infrared navigation. Moreover, theoretical analysis provides us a simple exponential function for the temperature distribution in the area around a corner. The presented investigation on temperature distribution in the area close to a corner is a new and original approach.

## 2. Theoretical Analysis Using a Variational Principle

The use of a variational principle, or more generally, variational calculus, is a technique which seems to be forgotten in present times. Nevertheless, it is the fundamental basis of the widely known finite element technique. Moreover, variational principles can also be used to obtain simple analytical approximations, as will be shown further on in this paper [19,20].

**Figure 4:**
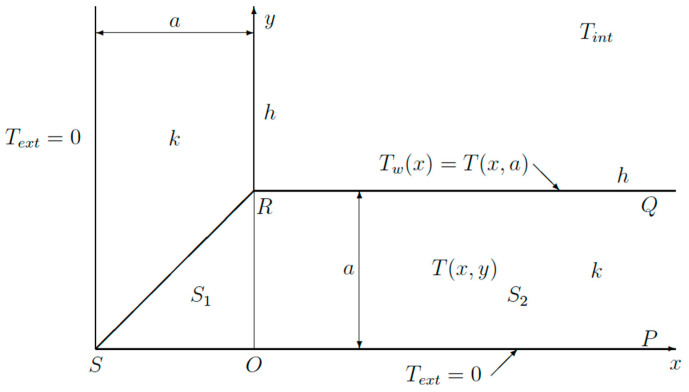
Schematic view of corner used for the theoretical analysis.

In order to obtain the temperature distribution along a wall, a simple thermal model will be used, as can be found in many textbooks [21,22,23,24,25]. It will be shown, at the end of this paper, that this approach will be effective for the experimental measurements. Figure 4 shows a schematic view of a corner composed of two walls.

The walls are made from a material with a thermal conductivity *k*. On the inside, we have convective cooling, *h* being the heat transfer coefficient. The air inside has a temperature *T_int_*. The part *PQ* of the wall has a temperature distribution *T_w_*(*x*), which has still to be determined. On the outside, the wall temperature is assumed to be exactly at the same temperature as the outside air *T_ext_*. As we are only interested in temperature rises, one can set *T_ext_ =* 0 without loss of generality. This isothermal boundary condition is mainly introduced to simplify the mathematical analysis.

Inside the wall, the temperature distribution *T*(*x*,*y*) satisfies the Laplace’ equation:

(1) $$\nabla^{2}T=0$$

Due to symmetry, one has just to consider one half of the geometry shown in Figure 4: the triangle *S*_1_ and the half infinite strip *S*_2_. At the bottom *SOP,* one has the boundary condition:

(2) T=0

On the internal side *RQ* convection has to be taken into account:

(3) $$k\frac{\partial T}{\partial y}+h\left(T_{int}-T\right)=0$$

In order to use the variational approach, one has to start from the following functional [19]:

(4) $$J\left(T\right)=\iint_{S_{1}\cup S_{2}}^{}{\left(\nabla T\right)}^{2}dxdy+\frac{h}{k}\int_{RQ}^{}{\left(T-T_{int}\right)}^{2}dx$$

where *S*_1_ denotes the triangular area *ORS* and *S*_2_ the area *QROP*. It can be proved that the functional (4) will be extremum for a function *T*(*x*,*y*), which satisfies (1), (2) and (3) [19]. If we insert an approximate function for *T*(*x*,*y*) in (4), the extremum value for J(T) will not be obtained. In our analysis, we will propose an approximate solution for the temperature distribution, which still includes two parameters. The extremization of the functional afterwards will then provide the best estimations for these parameters.

At a large distance from the corner (*x→∞*), one has the well-known one-dimensional solution:

(5) $$T_{w}\left(\infty \right)=\frac{T_{int}}{1+k/ha}$$

(6) $$T\left(\infty ,y\right)=\frac{T_{int}}{1+k/ha}\frac{y}{a}$$

To extremize the functional, we propose the following temperature distribution along *RQ*:

(7) $$T_{w}\left(x\right)=\frac{T_{int}}{1+k/ha}+∆Te^{-x/L}$$

where *ΔT* and *L* are two unknown parameters. This expression has been chosen because it may represent a temperature decreasing towards the corner. Inside the wall *S_1_*, the following test function is used:

(8) $$T\left(x,y\right)=\frac{\left(x+a\right)y}{a^{2}}[\frac{T_{int}}{1+\frac{k}{ha}}+∆T]$$

Inside the wall *S*_2_, we use:

(9) $$T\left(x,y\right)=\frac{y}{a}[\frac{T_{int}}{1+\frac{k}{ha}}+\;∆Te^{-\frac{x}{L}}]$$

One can easily verify that (8) and (9) satisfy the boundary conditions (2) and (3). Equations (8) and (9) are also continuous along the line *OR* or *x = 0*. As already pointed out, the proposed test function (8) and (9) need not satisfy Equation (1), and that is just the strength of the variational approach.

Inserting (8) and (9) in the functional (4) gives rise to the following functional after a long calculation:

(10) $$J=∆T^{2}-\left[\frac{1}{3}+\frac{L}{2a}+\frac{a}{6L}+\frac{hL}{2k}\right]+∆T\frac{2}{3}\frac{T_{int}}{1+\frac{k}{ha}}\ldots$$

where the terms not containing *ΔT* or *L* were omitted as they are not used further on. Now we have to extremize the functional *J* by writing:

(11) $$\frac{\partial J}{\partial L}=\frac{\partial J}{\partial ∆T}=0$$

Solving (11) yields:

(12) $$\frac{L}{a}=\frac{1}{\sqrt{3\left(1+ha/k\right)}}$$

and:

(13) $$∆T=-T_{int}\frac{\frac{ha}{k}}{1+\frac{ha}{k}}\;\frac{1}{1+\sqrt{3\left(1+\frac{ha}{k}\right)}}$$

**Figure 5:**
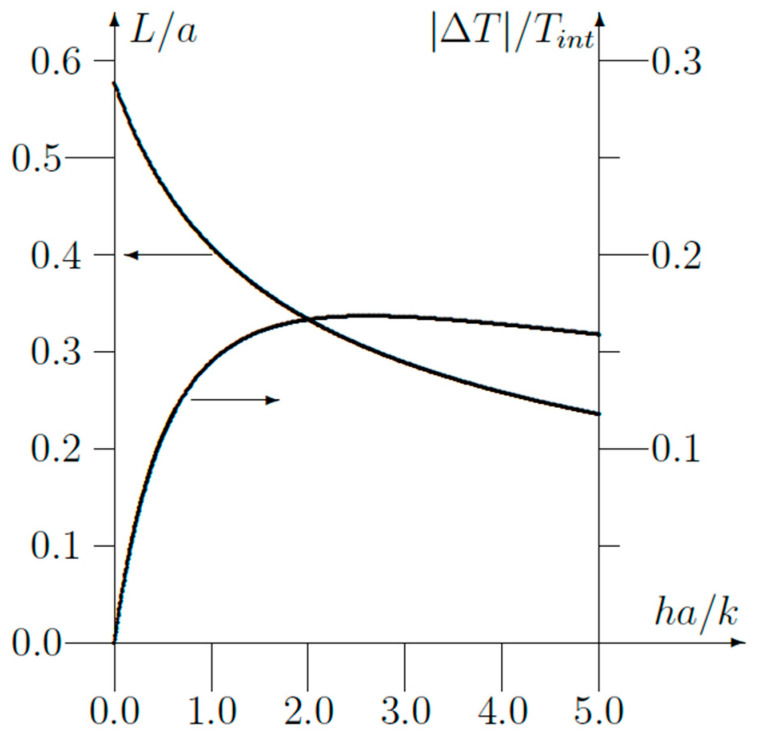
Plot of the parameters *L*/*a* and $\left|∆T\right|/T_{int}$ as functions of *ha/k* obtained from the theoretical analysis.

Equations (12) and (13) are two simple expressions giving the length constant *L* and the temperature drop *ΔT* near a corner. A plot of *ΔT/T_int_* and *L/a* functions of the dimensionless parameter *ha*/*k* is shown in Figure 5. Both parameters are easy to determine using a thermographic camera, as will be explained further on.

## 3. Solution with Another Test Function

**Figure 6:**
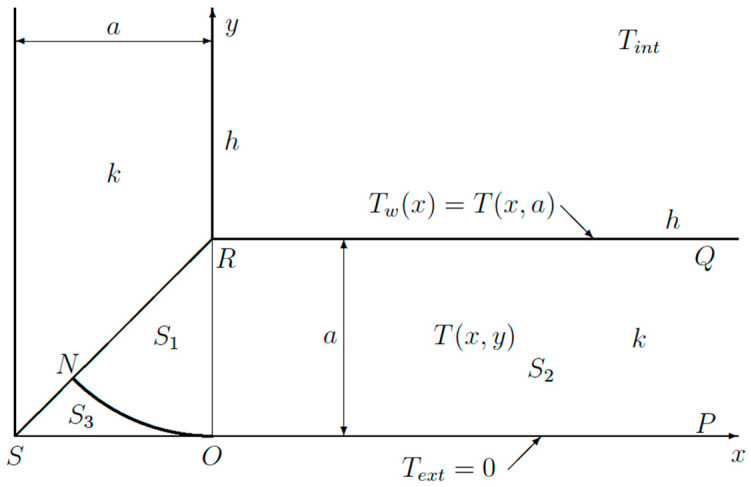
Schematic view of corner used for the analysis with a different test function.

In order to prove the general validity of the variational approach, another test function instead of (9) will be used (Figure 6). 

The triangular domain *SOR* is now split by a circular arc into two domains: *S*_1_ and *S*_3_ (Figure 6). The border in between is a circular arc *ON* with radius *a* and center point *R*. Inside *S*_1_, the test function varies linearly between point *R* and any point on the border *ON*. Hence, the temperature gradient is just a constant so that:

(14) $$\left|\nabla T\right|=\frac{T_{int}}{1+\frac{k}{ha}}\;1/a$$

which can be inserted into the functional (4). In the domain *S*_3_, the test function and, hence, the gradient is zero. In *S*_2_, the same function (9) is still used. 

Mathematical details will be omitted here because the calculation is analogous. The functional *J* turns out to be given by:

(15) $$J=∆T^{2}\;\left[\frac{\pi }{8}+\frac{L}{2a}+\frac{a}{6L}+\frac{hL}{2k}\right]+∆T\frac{\pi }{4}\frac{T_{int}}{1+\frac{k}{ha}}\ldots$$

Extremization of the functional *J* gives the same result (12) for *L/a*. Concerning Δ*T*, we now get:

(16) $$∆T=-\frac{3\pi }{8}T_{int}\frac{\frac{ha}{k}}{1+\frac{ha}{k}}\;\frac{1}{\frac{3\pi }{8}+\sqrt{3\left(1+\frac{ha}{k}\right)}}$$

Taking into account that 3*π*/8 *=* 1.178 is close to unity, it is clear that choosing a different test function has a minor influence on the final results. This proves the general applicability of the variational approach.

## 4. Extension to Non-Rectangular Corner

**Figure 7:**
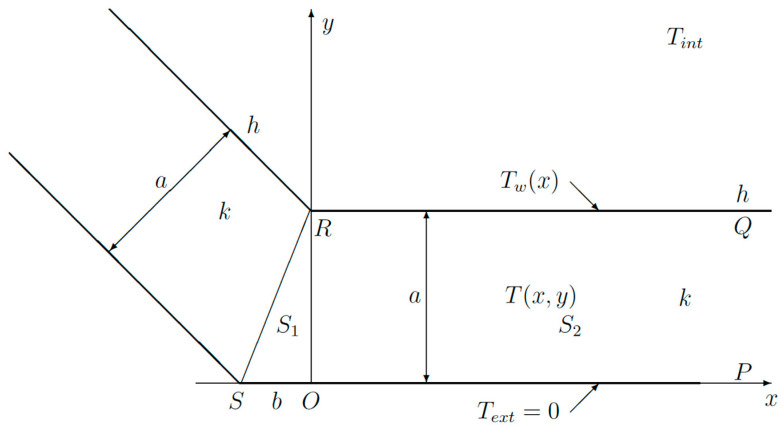
Schematic view of corner used for the theoretical analysis.

In Figure 7, a more general case has been drawn. A similar analysis can still be performed. Equations (1)–(7) and (9) remain valid. Equation (8) now has to be replaced by:

(17) $$T\left(x,y\right)=\frac{\left(x+b\right)y}{ab}\;[\frac{T_{int}}{1+\frac{k}{ha}}+∆T]$$

The functional *J* is now a bit more complicated:

(18) $$J={∆T}^{2}\left[\frac{b}{\;4a}\left(1+\frac{a^{2}}{3b^{2}}\right)+\frac{L}{2a}+\frac{a}{6L}+\frac{hL}{2k}\right]+∆T\frac{2T_{int}}{3\left(1+k/ha\right)}+\ldots$$

After extremizing the functional *J* by solving (11), one gets the same expression as (12) for *L*/*a*. The temperature drop Δ*T* is now:

(19) $$∆T=-T_{int}\frac{\frac{ha}{k}}{1+\frac{ha}{k}}\;\frac{1}{\frac{b}{4a}\left(3+\frac{a^{2}}{b^{2}}\right)+\sqrt{3\left(1+\frac{ha}{k}\right)}}$$

Obviously, for *b = a*, we obtain (13). For *b =* 0, *ΔT =* 0 as expected.

## 5. Numerical Simulations

There are two reasons for a numerical simulation to be considered mandatory. First of all, the variational approach presented in the foregoing section is an approximate method. Hence, the accuracy of the analytical results must be verified. Furthermore, the analytical approach assumes the isothermal boundary condition at the outer side of the walls. With the numerical simulation, convection at both sides will be taken into account. 

**Figure 8:**
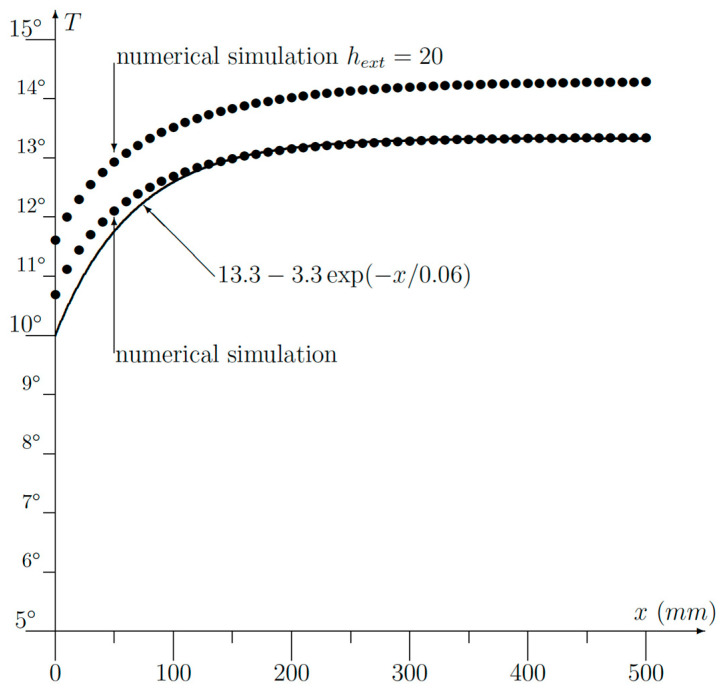
Numerical results obtained with *h* = 10, *k* = 1 and *a* = 0,2 compared with the analytical approximation. The upper curve is a numerical simulation with convection at the outside taken into account (*h_ext_ =* 20 W/m^2^K).

A program has been written in MATLAB to solve the problem numerically using the finite difference approach. The entire geometry was discretized in small squares of 1 cm *×* 1 cm. First of all, the numerical simulation was used to check the accuracy of the analytical approximation (13). For a wall thickness of *a =* 0.2 m, a thermal conductivity *k =* 1 W/mK and a heat transfer coefficient *h =* 10 W/m^2^K were considered. The inside and outside temperatures were *T_int_ =* 20 °C and *T_ext_ =* 0 °C, respectively. The numerical results are shown in Figure 8, along with the analytical approximation (9). Using the same set of parameters, the values we got from (11) and (12) were *L =* 0.0666 m and ΔT = −3.333 K.

In the corner point *x =* 0, the analytical approximation provided 10 °C, whereas we obtained 10.67 °C or an absolute error of 0.67 °C. Normalization to the maximum temperature being 20 °C gives a relative error of 0.67/20 *=* 3.35%. 

For the sake of completeness, a simulation with convection at the external wall *SOP* has been performed (Figure 8). The heat transfer coefficient *h_ext_ =* 20 W/m^2^K was used to account for the wind speed outside the building. As expected, the temperature on the inside is somewhat higher, but the shape is very similar to the other ones.

## 6. Experimental Verifications

In our experiments, a wall made from bricks without any air gap inside was used for the measurements. The wall has a thickness of *a =* 0.32 m. The air temperature inside was 24.5 °C. The air outside was found to be 7.8 °C. The temperature distribution along the wall was measured with an AGEMA 9000 thermographic camera. Both air temperatures were also measured using the same camera by hanging a sheet of paper in the air for a sufficiently long time. Measurements were done during a day without any notable sunshine.

**Figure 9:**
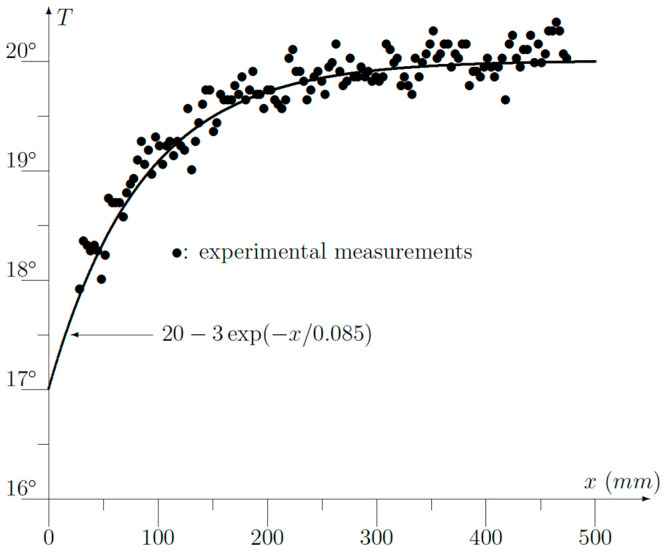
Experimental measurements obtained via infrared thermography.

The experimental results are displayed in Figure 9. *x* is the distance measured from the corner. First of all, the experimental points could be very well fitted to an exponential function like (7) using *L =* 85 mm and Δ*T* = −3 °C.

If we use the data: *h =* 10, *k =* 1 and *a =* 0.32 m, we obtain from (12):

(20) $$L=\frac{a}{\sqrt{3\left(1+\frac{ha}{k}\right)}}=0.0901\;m$$

which agrees very well with the experimental value of 85 mm. To find the temperature drop Δ*T*, we must use *T_int_ =* 24.5 *−* 7.8 *=* 16.7 °C because the theoretical analysis used *T* = 0 °C as the reference temperature. Then, we get:

(21) $$∆T=-T_{int}\frac{\frac{ha}{k}}{1+\frac{ha}{k}}\;\frac{1}{1+\sqrt{3\left(1+\frac{ha}{k}\right)}}=-2.79\;{}^{\circ}C$$

which also agrees quite well with the experimental value found to be 3 °C. A third verification can be found by checking (5):

(22) $$T_{w}\left(\infty \right)=\frac{T_{int}}{1+k/ha}=\frac{16.7}{1+\frac{1}{3.2}}=13.12\;{}^{\circ}C$$

But, in (5), the reference temperature was assumed to be zero. So, we have to add the outside temperature of 7.8 °C, which gives us 7.8 *+* 13.12 *=* 20.92 °C, which once again provides us a good agreement with the measured value of 20 °C (Figure 9). 

The good agreement between the theoretical formulae (5), (7), (12) and (13) and the experimental measurements proves, once again, the usefulness of the variational approach. Moreover, a simple formula like (7) is obtained so that an expensive or heavy computer is not required. The latter is certainly an advantage in the case of portable equipment.

## 7. Simulation Data in Various Scenarios

**Figure 10:**
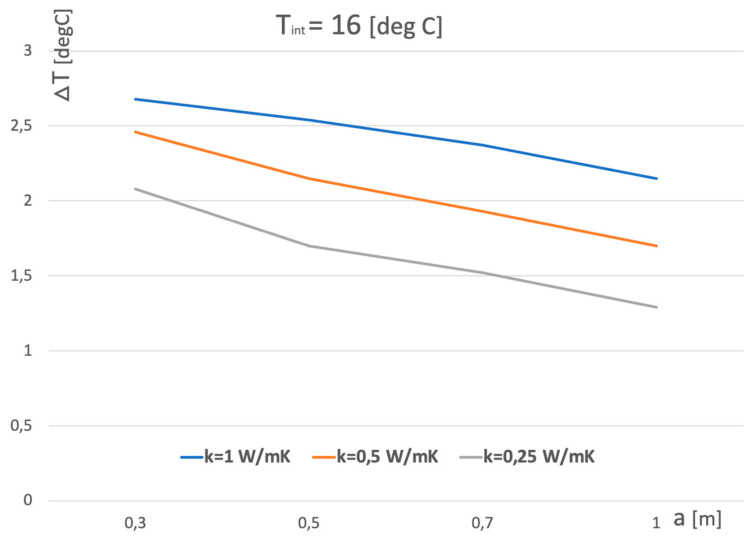
Corner temperature difference versus thickness of wall for various wall thermal conductivity when relative temperature *T_int_* (inside–outside) equals 16 °C.

**Figure 11:**
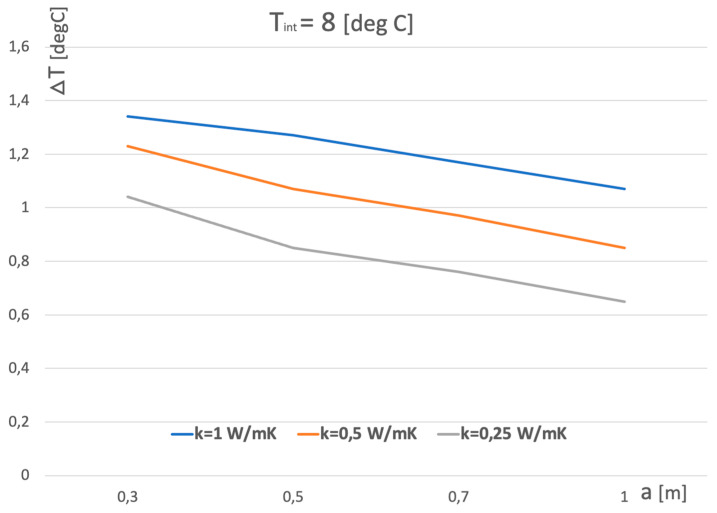
Corner temperature difference versus thickness of wall for various wall thermal conductivity when relative temperature *T_int_* (inside–outside) equals 8 °C.

Based on theoretical and experimental results, practical examples have been presented. Figure 10 and Figure 11 show the temperature dependence of ∆T on the wall thickness *a*. It is worth noting that for a very well thermally insulated wall with an average thermal conductivity of 0.25 W/mK, ∆T is greater than 0.6 °C, which is easy to recognize by an infrared camera with an average resolution.

**Figure 12:**
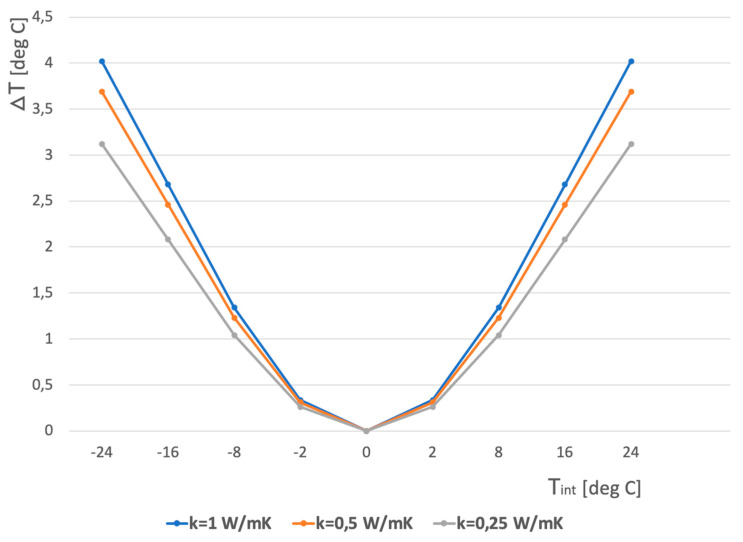
Corner temperature difference versus relative temperature *T_int_* (inside–outside) for various wall thermal conductivity.

Figure 12 shows that if the relative temperature *T_int_* is higher than 5 °C, a thermal resolution of 0.5 °C is sufficient to recognize the corner. A difference between indoor and outdoor temperatures of around 5 °C and more is normal during all seasons. Currently, one can find a cheap infrared camera with sufficient resolution on the market.

## 8. Discussion

Modern buildings are thermally well insulated. But, on the other hand, modern portable thermographic cameras have a high resolution, and 0.1 °C is no longer exceptional.

A non-homogeneous wall will give rise to a spatially fluctuating temperature. But modern thermographic cameras have a high spatial resolution as well so a curve fitting with a simple exponential function (7) is easily done. It must be pointed out here that the experimental results shown in Figure 9 have been done on a 70-year-old wall. The bricks contain several air cavities, and the mortar was not uniformly applied. Only the plaster on the inside was uniform. This explains the noisy behavior of the measurements (Figure 9), but nevertheless, a fitting with (7) was easily performed. 

The situation is similar in the case of new buildings that are mostly made of poured concrete with an insulating layer. The layer reduces the temperature difference. However, the high resolution of the infrared cameras allows us to notice the temperature difference in the corner of the wall. Considering the significant changes in the temperature of the outer wall of the building during the day and night, and the much more stable temperature conditions inside the building, the situation of equalizing the temperature in the corner with the temperature of the walls outside the corner occurs very rarely and is not a problem for the operation of our navigation system.

Here, it is worth emphasizing that the accuracy of the measurement is not required because the measurement is based on the temperature difference and not on its absolute value. In addition, the use of infrared instead of visible light automatically filters out any images that might interfere with the distance measurement. The use of ultrasonic or laser sensors requires a lot of energy to power the transmitters. Our method eliminates the need to use energy to power the transmitters.

This article presents an extension of our navigation system to recognize the approaching obstacle in the form of the end of the corridor. It allows people to recognize whether it is the end of the building or only its internal part. In the case of the internal corner, there will be no temperature difference, which is important information for a human or robot. Such an obstacle will be detected by our method using the difference in emissivity of floors and walls, which is described in our work [17].

## 9. Conclusions

The presented line of thought is an extension and continuation of our work described in [17]. The new idea leads to the detection of significant obstacles in the path of blind people in the form of a corner wall ending the corridor. This will significantly facilitate the navigation for blind and visually impaired people and mobile robots moving around corridors of public buildings. In our previous work, we described the navigation mechanism that uses the differences in emissivity of the materials used to build the walls and floors of the corridor. In this work, we use the same phenomenon of emission in the infrared band, taking into account the temperature distribution caused by the change in the geometry of the walls at the corners. It should also be emphasized that, in this work, we use the phenomenon of free heat radiation by the environment, which significantly reduces the amount of energy used for measurements. In addition, the hardware used for navigation is the same as in the previous solution. This solution simplifies the hardware aspect and helps save energy used for mobile navigation. Using both solutions, we significantly facilitate the movement of blind people and robots in the corridors of buildings where satellite navigation signal (e.g., GPS) is very limited or absent. In this work, theoretical analyzes were experimentally verified using the AGEMA 9000 thermographic camera, and we showed that it is possible to identify a corner obstacle with an accuracy of not worse than 5.5 cm.

It has also been proved that a simple exponential function can be used effectively for wall temperature measurement. 

## Data Availability

MDPI Research Data Policies.
